# Advances in Non-Invasive Myocardial Stiffness Assessment and Clinical Applications in Hypertrophic Cardiomyopathy

**DOI:** 10.31083/RCM47459

**Published:** 2026-03-09

**Authors:** Yipeng Gao, Nan Qi, Hao Gao, Youbin Deng, Yani Liu

**Affiliations:** ^1^Department of Medical Ultrasound, Tongji Hospital, Tongji Medical College, Huazhong University of Science and Technology, 430030 Wuhan, Hubei, China; ^2^Research Center for Mathematics and Interdisciplinary Sciences, Shandong University, 266237 Qingdao, Shandong, China; ^3^Frontiers Science Center for Nonlinear Expectations, Ministry of Education, 266237 Qingdao, Shandong, China; ^4^School of Mathematics and Statistics, University of Glasgow, G12 8QQ Glasgow, Scotland, UK

**Keywords:** shear wave, intrinsic velocity propagation, myocardial stiffness, hypertrophic cardiomyopathy

## Abstract

Myocardial stiffness is a central determinant of diastolic dysfunction and clinical outcomes in hypertrophic cardiomyopathy; however, a non-invasive evaluation of myocardial stiffness remains challenging. Emerging techniques, such as elastography, offer direct, non-invasive quantification of myocardial stiffness, providing critical insights beyond conventional indirect surrogates. This review summarizes the principles, validation, and clinical evidence for current non-invasive techniques for assessing myocardial stiffness. We further discuss the clinical applications of these techniques in hypertrophic cardiomyopathy, including diagnostic refinement, fibrosis detection, risk stratification, and treatment monitoring, as well as the challenges and future directions required for broader clinical translation. Ultimately, the non-invasive assessment of myocardial stiffness holds promise for transforming patient management to phenotyping and therapeutic decision-making through a more precise, mechanism-based approach.

## 1. Introduction

Hypertrophic cardiomyopathy (HCM) is a genetic cardiomyopathy characterized by myocardial hypertrophy that is unexplained by abnormal loading conditions [1]. Substantial progress has been made in preventing sudden cardiac death [2, 3] and managing left ventricular outflow tract obstruction [4]. However, heart failure (HF) remains a major challenge [5]. Myocardial stiffness is the core physical determinant of ventricular diastolic filling and compliance, which is the fundamental cause of HF syndromes [6].

Despite its importance, non-invasive evaluation of myocardial stiffness remains challenging. Conventional markers such as the New York Heart Association functional class, natriuretic peptides, and standard echocardiographic indices like the E/e^′^ ratio are indirect surrogates [7, 8]. These markers primarily reflect the downstream hemodynamic consequences of increased stiffness. Furthermore, they often lack the sensitivity to detect early or subtle changes in the myocardium itself [9].

In recent years, advanced imaging modalities have emerged to address this unmet need. Shear wave elastography, magnetic resonance elastography, and intrinsic cardiac elastography, each provide novel approaches for assessing myocardial stiffness. These techniques offer the potential for more direct and quantitative assessment [10, 11]. Nevertheless, all of these techniques have important limitations, including technical complexity, restricted availability, and lack of methodological standardization. These limitations currently hinder their routine clinical application.

This review provides a comprehensive overview of current non-invasive techniques to assess myocardial stiffness. We summarize their principles, validation against reference standards, and relative advantages and limitations. We further explore their emerging clinical applications in HCM, aiming to clarify their translational potential for improving diagnosis, risk stratification, and monitoring treatment.

## 2. Principles of Non-Invasive Assessment of Myocardial Stiffness 

### 2.1 Defining the Target: Myocardial Stiffness

When evaluating cardiac mechanics, it is essential to distinguish between chamber stiffness and myocardial stiffness. Chamber stiffness reflects the macroscopic mechanical behavior of the entire cardiac chamber, with pressure-volume loop analysis serving as the clinical gold standard [12]. In contrast, myocardial stiffness represents the intrinsic material property of the myocardial tissue itself, typically assessed under laboratory conditions by subjecting explanted myocardial strips to uniaxial stretching and recording the stress-strain relationship [13]. Recent technological advances have enabled the non-invasive measurement of myocardial stiffness (Table 1, Ref. [14, 15, 16, 17, 18, 19, 20, 21, 22]). What is directly measured is typically the velocity of mechanical waves, which has a specific physical relationship with myocardial stiffness (elastic modulus, kPa).

**Table 1.** **Non-invasive assessment of myocardial stiffness in hypertrophic cardiomyopathy**.

Study	Technique	Measurements	Study subjects	Main findings
Benz *et al*., 2025 [22]	Intrinsic cardiac elastography	iVP (m/s)	16 healthy volunteers vs. 10 HCM vs. 28 cardiac amyloidosis	(1) similar between HCM and healthy; (2) correlated with diastolic and fibrosis parameters
Naser *et al*., 2021 [14]	Intrinsic cardiac elastography	iVP (m/s)	30 controls vs. 51 HCM patients	(1) faster in HCM; (2) correlated with myocardial fibrosis and disarray; (3) independent predictor of adverse events
Petrescu *et al*., 2025 [21]	Shear wave echocardiography	Mitral SWV (m/s)	37 healthy volunteers vs. 30 heart transplanted patients vs. 22 HCM patients	(1) faster with replacement fibrosis; (2) correlated with fibrosis parameters; (3) good performance in fibrosis classification
Strachinaru *et al*., 2019 [19]	Shear wave echocardiography	Natural SWV (m/s)	45 healthy volunteers vs. 43 HCM patients	(1) faster in HCM; (2) correlated with diastolic parameters; (3) good diastolic performance
Strachinaru *et al*., 2019 [16]	Intrinsic cardiac elastography	iVP (m/s)	42 healthy volunteers vs. 33 HCM patients	(1) non-constant in HCM; (2) faster in thicker myocardial wall
Strachinaru *et al*., 2020 [20]	Shear wave echocardiography	Aortic SWV (m/s)	10 healthy volunteers vs. 10 HCM vs. 10 HCM with septal reduction therapy	Local increase in aortic SWV at scar tissue
Villemain *et al*., 2018 [18]	Shear wave echocardiography	ARFI myocardial stiffness (kPa)	28 healthy children vs. 28 HCM children	(1) higher in HCM; (2) highest with restrictive physiology; (3) correlated with reduced exercise capacity
Villemain *et al*., 2019 [17]	Shear wave echocardiography	ARFI myocardial stiffness (kPa)	60 healthy volunteers vs. 20 HCM-HFpEF patients	(1) higher in HCM; (2) correlated with diastolic and fibrosis parameters; (3) good diastolic performance
Zhao *et al*., 2024 [15]	Magnetic resonance elastography	Myocardial stiffness (kPa)	1 HCM case	(1) higher than reference; (2) matched with fibrosis regions

ARFI, Acoustic radiation force impulse; HCM, hypertrophic cardiomyopathy; HFpEF, heart failure with preserved ejection fraction; iVP, intrinsic velocity propagation; SWV, shear wave velocity.

### 2.2 Shear Wave Elastography

#### 2.2.1 Physical Basis

Measuring shear wave velocity (SWV) allows for a direct, quantitative estimation of myocardial stiffness. The velocity (c) is fundamentally related to the tissue’s intrinsic stiffness (shear modulus, µ) and its density (ρ) by the formula Eqn. 1, and µ can be approximated to the clinically familiar Young’s modulus (E ≈ 3µ). Shear waves can be generated externally by acoustic radiation force, allowing for precise control but limiting measurements to a few centimeters [23]. Alternatively, natural shear waves, created by events such as valvular closure, yield a higher signal-to-noise ratio, though their timing and location are less controlled [24].

(1) $$\mu =\rho \,c^{2}$$

#### 2.2.2 Preclinical Validation

In animal models, shear wave elastography-derived SWV correlates strongly with myocardial stiffness measured via the end-diastolic pressure-volume relationship [25, 26] and *ex vivo* stress-strain testing [27, 28], all of which have confirmed its physiological relevance. However, direct validation of shear wave elastography against human myocardial stiffness using invasive pressure-volume analysis or *ex vivo* tissue testing is still lacking, representing a key limitation for translational application.

### 2.3 Intrinsic Cardiac Elastography

#### 2.3.1 Physical Basis

Intrinsic cardiac elastography quantifies myocardial stiffness by measuring the propagation velocity of a myocardial stretch wave generated by atrial contraction [29, 30]. Because this method depends on atrial contraction to generate the stretch wave, it cannot be applied in patients with atrial fibrillation, which is a clinically important limitation [31]. The relation between myocardial stiffness (E) and wave velocity (c) can be approximated by the Moens-Korteweg equation: c = √ (Eh / 2ρR), where h is the wall thickness, R is the ventricular radius, and ρ is the myocardial density [29]. This technique is accessible using high-frame-rate tissue Doppler imaging (>250 frames/s) on commercial scanners [11, 32].

#### 2.3.2 Validation With Gold Standard

Validation studies have provided encouraging evidence. In animal models, this propagation velocity showed strong correlation with *ex vivo* measurements of myocardial stiffness [29], confirming its physiological basis. In humans, the propagation velocity has been shown to correlate with non-invasive indices of chamber stiffness [11], and with histopathological alterations, including myocardial fibrosis and myocyte disarray [14]. However, direct validation of intrinsic velocity propagation (iVP) against myocardial stiffness in humans using invasive pressure-volume analysis or tissue testing is still lacking, highlighting a key gap for translational application.

### 2.4 Magnetic Resonance Elastography

#### 2.4.1 Physical Basis

Magnetic resonance elastography directly quantifies myocardial shear modulus by imaging low-frequency shear waves, generated externally by a mechanical actuator. Phase-contrast magnetic resonance imaging sequences capture the wave propagation, and the tissue stiffness is calculated from the wave velocity using Eqn. 1 [33, 34]. Cardiac magnetic resonance elastography remains technically challenging due to cardiac and respiratory motion, the thin and anisotropic ventricular wall, and the need for high spatial resolution and adequate signal-to-noise ratio [35].

#### 2.4.2 Preclinical Validation

Animal experiments have demonstrated that cardiac magnetic resonance (CMR) elastography can detect dynamic changes in myocardial stiffness across the cardiac cycle and differentiate normal from stiffened myocardium [36]. In humans, cardiac magnetic resonance elastography has only been reported in isolated case studies, which demonstrated its feasibility [15]. Overall, this technique remains largely preclinical or exploratory, with limited availability, high technical demands, and lack of standardized acquisition protocols.

### 2.5 Common Limitations of Techniques

Despite their promise, current non-invasive techniques to assess myocardial stiffness face several important limitations. First, detecting mechanical waves requires extremely high temporal resolution. Although ultrafast ultrasound imaging techniques have been developed to address this issue [37], their clinical validation and widespread adoption remain at an early stage. Magnetic resonance elastography faces similar challenges in terms of technical demand and limited availability.

Second, wave propagation in the myocardium is more complex than initially assumed. Externally generated shear waves have high frequencies, attenuate rapidly, and typically propagate only a few centimeters [23]. In contrast, intrinsically generated low-frequency mechanical waves have longer wavelengths and are strongly influenced by cardiac geometry, making interpretation less straightforward [24].

Finally, myocardial stiffness varies at different points during the cardiac cycle. Systolic stiffness appears to depend largely on active myocardial properties such as contractility, whereas passive diastolic stiffness is more influenced by tissue composition and structural remodeling [10]. This distinction is critical for interpreting elastography results from different techniques. For example, intrinsic cardiac elastography, which measures stiffness following atrial contraction, primarily reflects passive matrix remodeling [16]. In contrast, shear wave elastography can assess stiffness throughout the cardiac cycle. Measurements at end-diastole reflect intrinsic elastic properties, while systolic measurements are strongly influenced by myocardial contractility [38]. The relationship between wave velocity derived from externally induced and intrinsically generated mechanical waves is also complex. A recent study demonstrated that the two measures show similar values at the same time points in healthy individuals, but they diverge significantly under pathological conditions such as HCM [39]. Therefore, interpretation of wave velocity should be approached with caution across different physiological and disease states, and further studies are needed to elucidate the underlying cardiac physiology beyond wave velocity measurements.

### 2.6 Summary and Critical Comparison

In summary, the three techniques differ markedly in methodological principles, reproducibility, and readiness for clinical use. Shear wave elastography measures propagation velocity induced by acoustic radiation force or natural shear waves. It provides estimates of quantitative stiffness with good reproducibility (intraclass correlation coefficients [ICCs]: 0.77–0.90) [17, 40] but depends on operator skill and acoustic window quality. Intrinsic cardiac elastography measures atrial contraction-driven stretch waves, offering high reproducibility in sinus rhythm (ICCs: 0.91–0.95) [14] and correlation with histopathology, yet it cannot be applied in atrial fibrillation. Magnetic resonance elastography derives three dimensions (3D) stiffness maps from externally induced shear waves, achieving excellent reproducibility (ICCs: 0.92–0.96) [41] but with low feasibility due to complex setup, motion sensitivity, and cost.

Ultrasound-based methods (shear wave elastography and intrinsic cardiac elastography) are currently the most feasible for clinical application, while magnetic resonance elastography remains largely confined to specialized research centers. All three have been validated in animal models against measurements of invasive or *ex vivo* stiffness. In humans, intrinsic cardiac elastography shows the strongest validation through correlations with histopathology and indices of non-invasive chamber stiffness. A concise comparison of their key features is summarized in Table 2 (Ref. [11, 13, 14, 17, 25, 26, 27, 28, 29, 36, 40, 41]).

**Table 2.** **Comparison of myocardial stiffness assessment techniques**.

Technique	Reproducibility	Clinical feasibility	Validation status
Shear wave echocardiography	Good: (1) 2D; (2) operator and acoustic window dependent; (3) ICC: 0.77–0.90 [17, 40]	Moderate: (1) commonly experiment scanner; (2) low cost	Animal models: (1) invasive pressure-volume analysis; (2) stress–strain testing [25, 26, 27, 28]
Intrinsic cardiac elastography	Good to excellent: (1) 2D; (2) operator and acoustic window dependent; (3) ICC: 0.91–0.95 [14]	High: (1) commercial scanner; (2) low cost	Animal models: stress–strain testing [29] Human: (1) histopathological alterations; (2) non-invasive end-diastolic pressure-volume relationship [11, 14]
Magnetic resonance elastography	Excellent: (1) 3D; (2) operator independent acquisition; (3) ICC: 0.92–0.96 [41]	Low: (1) special hardware and software; (2) high cost and time consumption	Animal models: (1) invasive pressure-volume analysis; (2) stress-strain testing [13, 36]

2D, two dimensions; 3D, three dimensions; ICC, intraclass correlation coefficients.

## 3. Mechanisms of Myocardial Stiffness in HCM

**Fig. 1.**
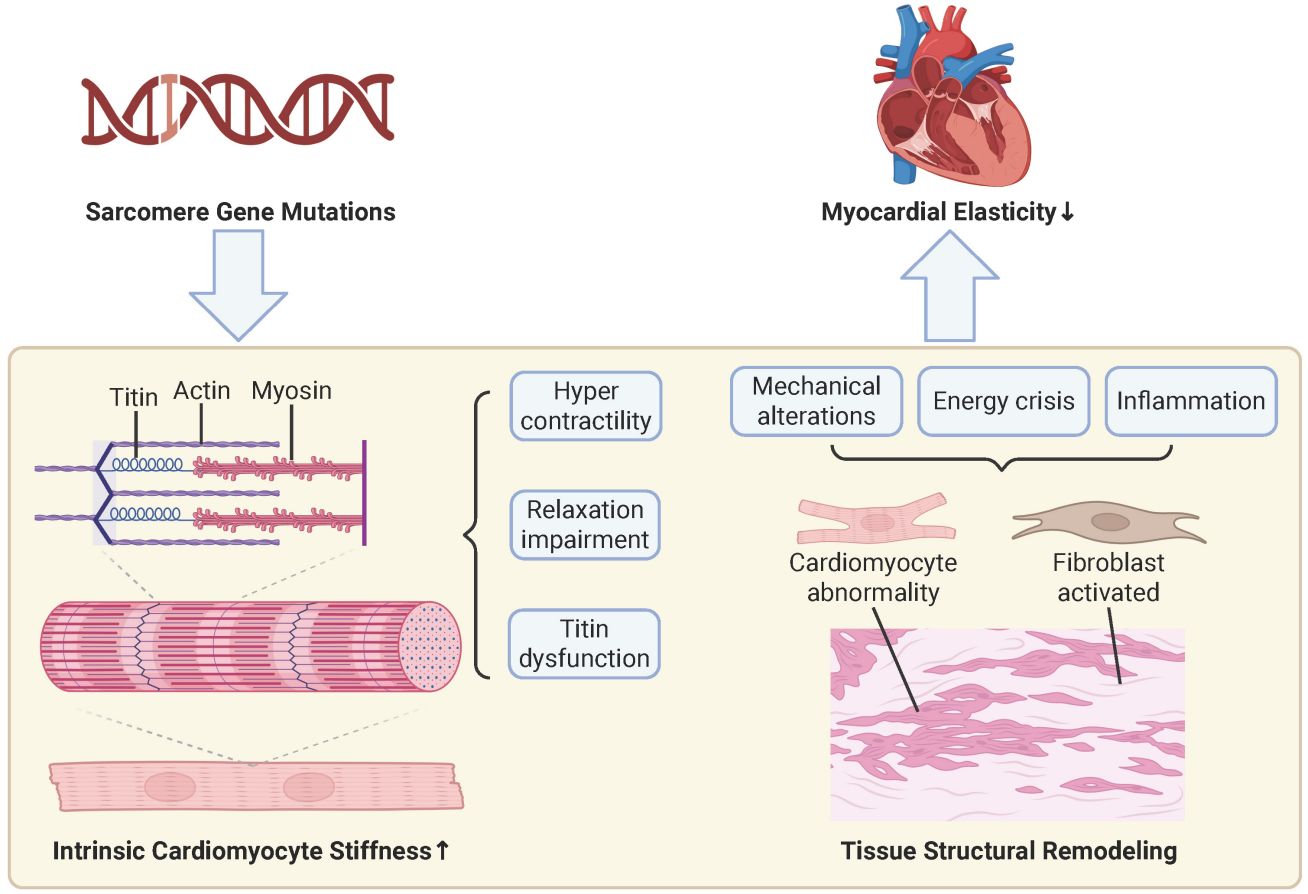
**Mechanisms of increased myocardial stiffness**. At the cellular level (left pathway), sarcomere gene mutations serve as the initiating factor, leading to sarcomere hypercontraction, impaired relaxation, and titin dysfunction, which collectively increase intrinsic cardiomyocyte stiffness. At the tissue level (right pathway), these primary cellular issues, compounded by secondary stressors such as altered mechanical stress, energy crisis, and inflammation, result in cardiomyocyte abnormalities and fibroblast activation, ultimately resulting in myocardial disarray and fibrosis. Collectively, the increased intrinsic myocyte stiffness from the cellular pathway and the adverse structural remodeling from the tissue pathway contribute to increased myocardial stiffness. The upward arrow indicates an increase, and the downward arrow indicates a decrease. This figure was created with BioRender.com (BioRender Inc., Toronto, ON, Canada).

In HCM, the development of myocardial stiffness is a multiscale pathological cascade involving dynamic interactions from molecular and cellular to tissue levels (Fig. 1).

### 3.1 Molecular and Cellular Alterations

Sarcomeric protein mutations, primarily in myosin heavy chain 7 (*MYH7*) and encoding myosin-binding protein C, cardiac-type (*MYBPC3*), which account for 50–75% of genetically confirmed HCM cases [42], lead to a state of hypercontractility in the myocardium, increasing systolic tension but impairing diastolic relaxation due to incomplete cross-bridge detachment [43]. This hypercontractile state consumes excessive adenosine triphosphate, leading to an energy crisis that further limits active relaxation processes like Ca^2+^ reuptake [43]. In addition, the giant protein titin, which regulates passive stiffness [44], is affected by phosphorylation imbalances in HCM [45]. Reduced activity of softening kinases (e.g., Protein Kinase A, Protein Kinase G) [46, 47] and increased activity of stiffening kinases (e.g., Protein Kinase C alpha) [48] increase titin stiffness, increasing passive tension.

### 3.2 Tissue Structural Remodeling

Chronic hemodynamic load and metabolic-mechanical imbalances ultimately trigger irreversible structural remodeling of myocardial tissue, solidifying myocardial stiffness at the structural level [49]. The core pathological feature is myocardial fibrosis, including irreversible replacement scarring and potentially reversible diffuse interstitial fibrosis [50, 51]. Myocyte disarray is another hallmark pathological change in HCM that structurally exacerbates mechanical dysfunction [52]. The orderly alignment of cardiomyocytes is replaced by chaotic, interwoven patterns [53], disrupting tissue synergy, leading to inefficient force transmission, and further reducing diastolic efficiency [54].

## 4. Clinical Applications of Non-Invasive Techniques to Assess Myocardial Stiffness in HCM

### 4.1 Diagnosis and Phenotyping

Assessment of non-invasive stiffness is a powerful tool for diagnosing and characterizing HCM. Studies using both naturally occurring and externally induced shear waves have consistently shown that myocardial stiffness is significantly higher in HCM patients compared to healthy volunteers. Villemain *et al*. [17] identified a stiffness cutoff of 8 kPa, which distinguished adult HCM patients from healthy individuals with 100% specificity and 95% sensitivity. In pediatric HCM, Villemain *et al*. [18] also found that diastolic stiffness was significantly elevated, particularly in patients with a restrictive physiology. In a study by Strachinaru *et al*. [19], natural SWV was used to classify pathologic myocardium with 95% sensitivity and 90% specificity.

The diagnostic application of iVP, measured by intrinsic cardiac elastography, has revealed more nuanced findings. Strachinaru *et al*. [16] first reported that while average iVP may not differ significantly between HCM patients and healthy controls, a large subset of HCM patients (42%) exhibit a unique non-constant wave propagation pattern, characterized by initial slow velocity followed by rapid acceleration. This suggests iVP can identify distinct biomechanical phenotypes within the HCM population. While still an emerging technique, a case study in an HCM patient by Zhao *et al*. [15] demonstrated a left ventricular stiffness of 21.8 kPa, substantially higher than the normal range of 7.2–9.8 kPa.

Despite encouraging diagnostic performance, most studies remain single-center with small sample sizes. Cutoff values may vary across imaging platforms and lack standardization. Therefore, it should be noted that the reported cutoff values are not yet standardized and should be applied cautiously in clinical practice. Moreover, it remains unclear whether stiffness mapping can distinguish HCM subtypes (e.g., obstructive vs. non-obstructive, apical vs. septal) or provide genotype-specific diagnostic insights. Future research should address these gaps with larger, multicenter studies and systematic phenotyping.

### 4.2 Assessment of Myocardial Fibrosis

A key application of these techniques is the non-invasive detection and grading of myocardial fibrosis, a critical component of HCM pathology. Myocardial stiffness measured by shear wave elastography, as shown by Villemain *et al*. [17], has a significant, positive correlation with markers of fibrosis on CMR, including late gadolinium enhancement (LGE) and myocardial T1 pre-contrast values. Similarly, Strachinaru *et al*. [20] found that natural SWV could detect local stiffness changes corresponding to scar tissue in patients after septal reduction therapy. Petrescu *et al*. [21] further quantified this relationship and showed that SWV increases as fibrosis progresses from interstitial to replacement types. The study identified clinically useful thresholds. An SWV below 6.0 m/s effectively rules out fibrosis, while an SWV above 8.1 m/s can distinguish severe replacement fibrosis from interstitial fibrosis with 100% specificity [21].

Studies also suggest that iVP is a sensitive marker of the underlying fibrotic burden. Naser *et al*. [14] first demonstrated a direct correlation between iVP and histologically confirmed myocardial fibrosis and myocyte disarray. Benz *et al*. [22] found that iVP was normal in early-stage, non-fibrotic HCM, suggesting its value increases with fibrosis progression. Zhao *et al*. [15] reported a strong spatial correlation between areas of increased myocardial stiffness on magnetic resonance elastography and regions of fibrosis identified by CMR in a patient with HCM.

Although these findings are promising, shear wave elastography thresholds require validation across different platforms and populations. Most iVP studies are cross-sectional, limiting their ability to monitor longitudinal fibrotic progression. Magnetic resonance elastography, while theoretically advantageous, remains impractical for clinical adoption due to its increased technical demands. From a pathophysiological standpoint, the initial increase in myocardial stiffness can be driven by molecular and cellular alterations, such as sarcomere hypercontractility and titin dysfunction [43, 44], which may precede the development of significant, irreversible tissue-level fibrosis [49]. Therefore, it is plausible that a detectable rise in myocardial stiffness serves as an earlier biomarker of mechanical dysfunction than the anatomical changes captured by LGE or extracellular volume (ECV). This creates an opportunity for a more nuanced, integrated assessment to stage a patient’s disease. For example, a patient presenting with elevated myocardial stiffness but with normal ECV and negative LGE may represent an earlier, “myogenic dysfunction-dominant” stage of the disease, which will be further discussed in Section 5.2. This state might signify a critical window of opportunity where proactive therapies targeting sarcomeric function, such as myosin inhibitors [55], could be most effective in preventing the progression to established fibrosis. Conversely, high stiffness coupled with extensive LGE signifies advanced, irreversible structural remodeling where therapeutic goals may shift. Thus, stiffness imaging provides complementary functional information that could refine risk stratification and guide the timing of therapeutic interventions. However, few studies have tested whether stiffness imaging provides incremental value beyond established CMR fibrosis markers. Addressing these limitations will be critical to moving fibrosis assessment from research to clinical application.

### 4.3 Prognosis and Risk Stratification

Measuring myocardial stiffness can provide valuable prognostic information beyond traditional risk markers. In a study by Villemain *et al*. [18] on pediatric HCM patients, increased myocardial stiffness measured by shear wave elastography was strongly correlated with reduced exercise capacity, a key indicator of functional limitation and prognosis. Naser *et al*. [14] showed that an elevated global iVP (>3.0 m/s) independently predicted adverse clinical outcomes in patients with HCM. These findings suggest that iVP’s ability to track the fibrotic burden provides important prognostic information throughout the disease course.

Current prognostic data are derived from limited, mostly single-center cohorts, with no large-scale, prospective, or multicenter validation. Whether stiffness improves established risk prediction models remains unknown. Furthermore, clinically reliable cutoff values for prognosis have not been established, and pediatric versus adult data are fragmented. Established risk models, such as the European Society of Cardiology Sudden Death Risk Model [56], are primarily designed to predict sudden cardiac death and do not adequately capture the trajectory of HF, which is a major driver of morbidity and mortality in this population [5]. This is where myocardial stiffness assessment may provide its greatest incremental value. Since increased myocardial stiffness is the fundamental mechanical basis for diastolic dysfunction and HF symptoms [6], its quantification offers a direct window into the patient’s risk for the progression of HF. Therefore, integrating stiffness into clinical practice could involve creating a novel, HF-centric risk stratification pathway. Existing multiparametric scoring systems, such as Heart Failure Association – pretest assessment, Echocardiography & natriuretic peptide, Functional testing, Final etiology (HFA-PEFF) and Heavy, Hypertensive, atrial Fibrillation, Pulmonary hypertension, Elder, Filling pressure (H_2_FPEF) have shown potential value for HF risk stratification in patients with HCM [57]. These frameworks could be further refined by incorporating stiffness metrics in addition to established echocardiographic indices of diastolic function (e.g., E/e^′^ ratio, left atrial volume), circulating biomarkers of wall stress and congestion (e.g., N-terminal pro-B-type natriuretic peptide), and CMR-derived fibrosis parameters (e.g., LGE extent, ECV fraction). Future studies should evaluate whether integrating stiffness into multiparametric risk models can improve patient stratification and clinical decision-making.

## 5. Future Perspectives

While non-invasive myocardial stiffness assessment has shown significant promise, the field is still evolving. Future advancements are expected to refine the technology, integrate it with other imaging modalities for deeper phenotyping, and ultimately guide precision medicine in HCM.

### 5.1 Technical Improvements

Future technical advancements in myocardial elastography are aimed at improving accuracy, expanding clinical applicability, and addressing the inherent complexities of cardiac mechanics.

#### 5.1.1 Overcoming Dependency: From Doppler to Speckle-Tracking

Doppler-based techniques, such as shear wave elastography and intrinsic cardiac elastography, while offering high temporal resolution, are angle-dependent, although this can be mitigated by careful alignment [31]. The next step will be the development of robust, angle-independent tracking algorithms, most likely based on advanced speckle-tracking. The analysis workflow can be time-consuming; future refinements, including semiautomatic tracking, will be essential to facilitate clinical adoption.

#### 5.1.2 Spatial Advancements: From 2D to 3D/4D

The propagation of elastic waves in the myocardium is complicated by the active properties of muscle fibers, the tissue’s anisotropy due to complex fiber orientations, and the ventricle’s geometry [37]. These factors induce a complex wave propagation pattern in three dimensions, which complicates the direct relationship between measured wave velocity and tissue stiffness. Recent pioneering work has utilized four dimensions (4D) ultrafast ultrasound to study this natural wave propagation in 3D, allowing for better determination of wave excitation sources and direction [58]. While this represents a major leap forward, both the imaging sequences and post-processing algorithms require further optimization before 4D elastography can be applied on a large scale.

#### 5.1.3 Artificial Intelligence for Quantification of Myocardial Stiffness

Artificial intelligence (AI) is transforming myocardial stiffness assessment by enabling fully automated post-processing, which mitigates operator dependency and poor reproducibility of conventional strain and elastography methods. Deep learning models, such as U-Net for cardiac segmentation and optical flow networks for tissue motion tracking, allow fast, robust estimation of displacement and strain directly from standard CMR or ultrasound images [59]. End-to-end architectures can even reconstruct elasticity maps from raw data, bypassing error-prone intermediate steps [60]. This automation lays the foundation for standardized, scalable stiffness quantification in clinical practice.

AI also shows promise in accelerating physics-based approaches, such as finite element analysis combined with CMR. Finite element analysis infers stiffness through inverse problems but is often computationally intensive, operator-dependent, and sensitive to model assumptions [61, 62, 63, 64]. Deep learning offers a data-driven alternative to replace iterative optimization, improving computational efficiency, however, these methods remain exploratory [65]. More immediately translatable is the integration of AI with radiomics, which extracts quantitative texture features from routine images such as CMR cine or ultrasound B-mode to detect patterns associated with myocardial fibrosis [66]. For example, radiomics models based on non-contrast CMR cine have accurately predicted LGE [67], and ultrasound radiomics enables contrast-free, semi-quantitative fibrosis assessment, offering an alternative for patients ineligible for CMR [68, 69].

Despite these advancements, a significant challenge remains due to the scarcity of large, high-quality labeled datasets needed for model training and validation. Nevertheless, AI is undoubtedly a core driving force poised to propel the non-invasive assessment of myocardial stiffness into mainstream clinical practice.

### 5.2 Multimodality Imaging for Advanced Phenotyping

**Fig. 2.**
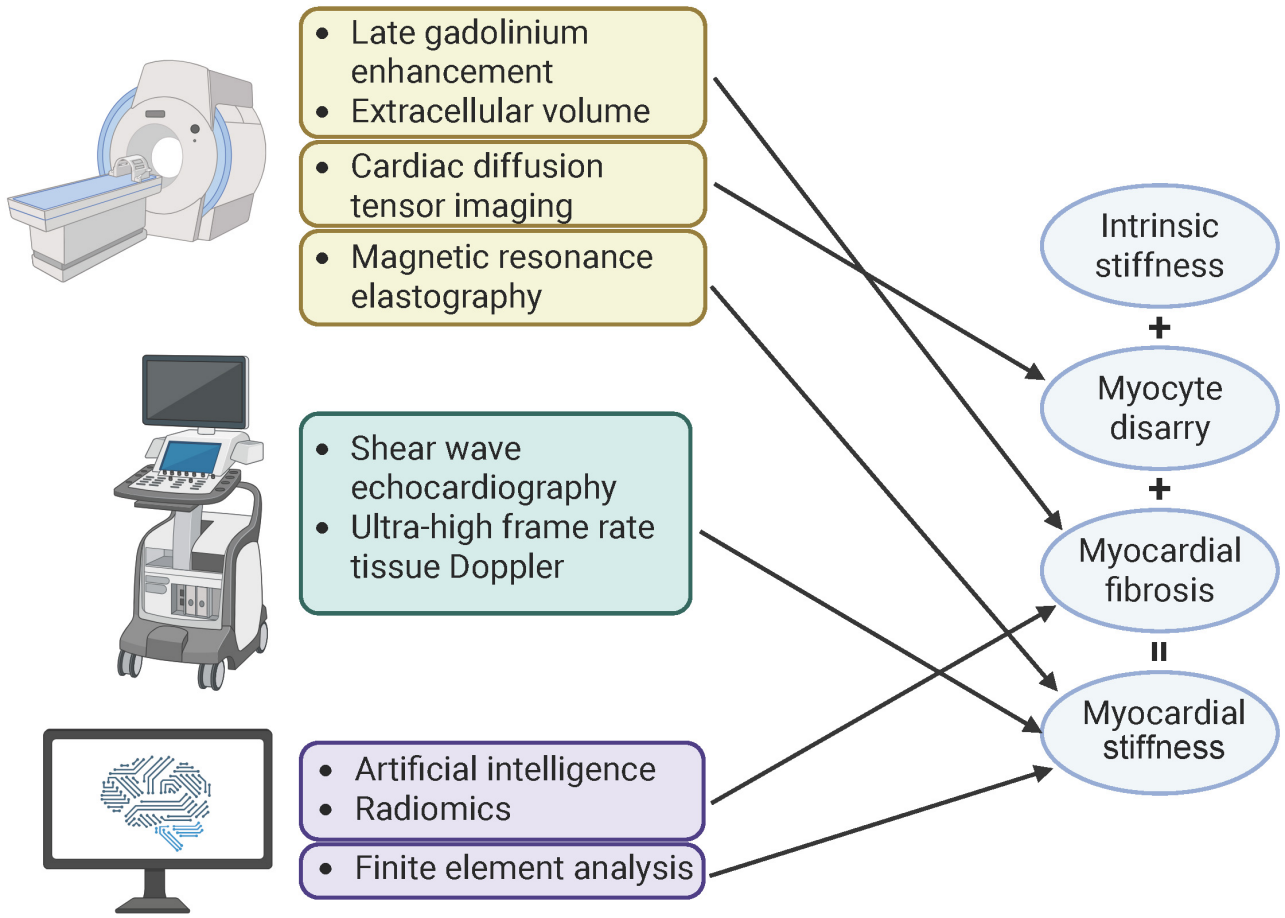
**Assessment of contributing factors for myocardial stiffness**. Myocardial stiffness arises from intrinsic stiffness, myocyte disarray, and myocardial fibrosis. Various techniques evaluate myocardial stiffness and its contributing factors. Magnetic resonance imaging provides detailed tissue characterization, using late gadolinium enhancement and extracellular volume to quantify myocardial fibrosis, and cardiac diffusion tensor imaging to assess myocyte disarray. Direct quantification of tissue stiffness can be achieved through magnetic resonance elastography, shear wave echocardiography, and intrinsic cardiac elastography. Additionally, artificial intelligence and radiomics analyze image features for characterization of myocardial fibrosis, while finite element analysis creates patient-specific biomechanical models to estimate overall myocardial stiffness. This figure was created with BioRender.com (BioRender Inc., Toronto, ON, Canada).

The true potential of myocardial stiffness measurement is realized when it is integrated with other imaging techniques to decode the specific contributors to stiffness (Fig. 2). The use of multimodality imaging holds promise for creating detailed “imaging phenotypes” of individual patients. Techniques such as LGE [70] and T1 mapping/ECV [71] provide core methods for assessing myocardial fibrosis, which is a primary structural source of increased myocardial stiffness [22]. Furthermore, breakthroughs have been achieved in assessing myocyte disarray using cardiac diffusion tensor imaging, an advanced technique that can depict the microstructural architecture of myocardial fiber bundles [72]. In a study by Naser *et al*. [14], the correlation of iVP with myocardial disarray suggests that the underlying causes of stiffness are themselves heterogeneous.

By integrating quantitative stiffness data from elastography with structural data from CMR, a more precise “myocardial stiffness phenotyping” can be developed. This would allow for the classification of patients into distinct subtypes, such as “fibrosis-dominant”, “myogenic dysfunction-dominant”, or “mixed” phenotypes. Identifying these dominant drivers could enable more precise risk stratification, identifying patients most likely to benefit from early, aggressive interventions.

### 5.3 Future Therapeutic Directions: Guiding Novel Therapies With Stiffness Phenotyping

While not yet part of routine clinical practice, emerging therapies hold promise for phenotype-guided management in HCM, with myocardial stiffness serving as a potential biomarker for patient stratification (Table 3, Ref. [73, 74, 75, 76]).

**Table 3.** **Hypertrophic cardiomyopathy novel therapies based on myocardial stiffness mechanisms**.

Drug	Target	Mechanism	Evidence	Prospects	References
Cardiac myosin inhibitors	Sarcomere hypercontraction	Allosteric inhibitor of myocardial myosin adenosine triphosphate enzyme, reducing contractile force and calcium sensitivity	Supported by positive results from multiple large trials	Improves pressure gradient, symptoms in obstructive, and exercise tolerance, quality of life in non-obstructive hypertrophic cardiomyopathy patients	[73, 74]
Mineralocorticoid receptor antagonists	Aldosterone pathway	Inhibits the aldosterone pathway, anti-fibrotic	Demonstrates anti-fibrotic effects in other heart failure types, with limited hypertrophic cardiomyopathy-specific randomized controlled trial data	Unclear role in hypertrophic cardiomyopathy; further research needed to confirm anti-fibrotic effects	[75]
Sodium-glucose cotransporter 2 inhibitors	Multiple downstream pathways	Promotes ketone body conversion, relieves energy crisis, inhibits sodium-hydrogen exchange, reduces intracellular sodium/calcium, anti-inflammatory, anti-oxidative, anti-fibrotic	Promising real-world signals; large randomized controlled trial results pending	Highly promising, potential foundational treatment for obstructive hypertrophic cardiomyopathy	[76]

#### 5.3.1 Targeting Sarcomere Hyperfunction

For patients with the “myogenic dysfunction-dominant” phenotype, cardiac myosin inhibitors, such as Mavacamten and Aficamten, are tailored for this phenotype [55]. These agents selectively stabilize myosin in a super-relaxed state, reducing the number of available myosin heads and thereby decreasing intrinsic myocardial contractility and calcium sensitivity [55]. Clinical trials (e.g., EXPLORER-HCM, VALOR-HCM) have confirmed that Mavacamten significantly reduces the E/e^′^ ratio and improves left atrial volume, indicators that reflect ventricular filling pressures and diastolic function [73, 74], suggesting attenuation of functional stiffness.

#### 5.3.2 Targeting Fibrosis

For fibrosis-dominant phenotypes, treatment should focus on slowing or reversing structural remodeling to prevent permanent loss of myocardial elasticity. Mineralocorticoid receptor antagonists, such as spironolactone, theoretically exert antifibrotic effects by inhibiting the aldosterone pathway. Although they are beneficial in other HF types [75], there is currently a lack of large-scale randomized controlled trials specifically evaluating the impact of mineralocorticoid receptor antagonists on myocardial fibrosis or clinical outcomes in HCM patients.

#### 5.3.3 Broad-Acting Therapy

Sodium-glucose cotransporter 2 inhibitors exhibit pleiotropic cardioprotective effects, including improving myocardial energy metabolism, inhibiting the sodium-hydrogen exchanger, and providing anti-inflammatory, anti-oxidative stress, and anti-fibrotic benefits [77, 78]. These mechanisms are highly consistent with the downstream pathological pathways of HCM. Real-world data suggest reduced mortality and hospitalizations in HCM patients [76], and ongoing trials (e.g., SOTA-CROSS HCM [NCT06433050], SONATA-HCM [NCT06481891]) will clarify their role in stiffness-informed therapeutic strategies.

## 6. Conclusions

Increased myocardial stiffness is a core pathophysiological feature of HCM, and is responsible for its diverse clinical manifestations [17]. Non-invasive assessment of myocardial stiffness has emerged as a promising frontier in cardiac imaging. This review summarizes the principles and validation of current technologies, while also acknowledging their limitations. Emerging clinical evidence highlights their potential to advance the management of HCM. These techniques not only facilitate diagnosis and phenotyping by identifying distinct stiffness patterns, but also provide crucial insights into myocardial fibrosis, risk stratification, and prognosis. By integrating stiffness measurements with complementary imaging modalities, a more refined “myocardial stiffness phenotyping” can be achieved, which holds the key to guiding novel therapies. Despite these advances, significant challenges remain. Future research must address the lack of technical standardization, validate findings in large multicenter cohorts, and establish clinically accurate thresholds for diagnosis and prognosis.
